# *Haemaphysalislongicornis* ticks are unable to transstadially transmit *Theileria haneyi* to horses

**DOI:** 10.3389/fvets.2025.1572944

**Published:** 2025-04-02

**Authors:** Karen C. Poh, Kennan Oyen, Cynthia K. Onzere, Lowell S. Kappmeyer, Reginaldo G. Bastos

**Affiliations:** ^1^Animal Disease Research Unit, USDA-ARS, Pullman, WA, United States; ^2^Department of Veterinary Microbiology and Pathology, College of Veterinary Medicine, Washington State University, Pullman, WA, United States

**Keywords:** *Theileria haneyi*, *Haemaphysalis longicornis*, Asian longhorned ticks, tick transmission, equine piroplasmosis, horses

## Abstract

**Introduction:**

The recent discovery of *Theileria haneyi*, a tick-borne hemoparasite that causes mild clinical signs of equine piroplasmosis, has added complexity to the diagnosis of this reportable disease, which is prevalent among equids globally. Knowledge gaps regarding competent tick vectors that can transmit *T. haneyi* and the recent outbreak of *Haemaphysalis longicornis* in the US has prompted us to conduct this study. Our objective was to investigate whether *H. longicornis* can transstadially transmit *T. haneyi* to horses.

**Materials and methods:**

*Haemaphysalis longicornis* larvae (0.5 g) and nymphs (*n* = 500) were fed on a splenectomized *T. haneyi*-infected horse for parasite acquisition. During the tick feeding period, parasitemia was monitored using nested PCR (nPCR) and blood smear analysis. The acquisition ticks fed until repletion and were transferred to an incubator for molting. Concomitantly, red blood cells (RBCs) were collected from the acquisition horse for further infection. Freshly molted nymphs (*n* = 282) and adults (*n* = 212), 22 offsprings of the acquisition larvae and nymphs, respectively, were placed on two individual naïve spleen-intact horses for transstadial parasite transmission. Another naïve horse was inoculated with 1 mL of RBCs from the acquisition horse. After tick infestation and RBC inoculation, the transmission horses were monitored for 38 days for the presence of *T. haneyi* DNA in their peripheral blood using nPCR, as well as for any clinical signs of infection.

**Results and discussion:**

The splenectomized acquisition horse developed canonical signs of acute *T. haneyi* infection during tick acquisition. The percentage of parasitized RBCs in the acquisition horse varied between 2.2 and 8.1% during the tick feeding stage. Out of a subset of 10 engorged larvae that fed on the acquisition horse, all ticks tested nPCR positive for *T. haneyi.* However, only 4 out of 10 engorged nymphs that fed on the acquisition horse tested PCR positive for *T. haneyi*. We found no evidence for the presence of parasite DNA in the transmission ticks or in the horse’s blood nor did we observe any clinical signs of *T. haneyi* infection in the transmission horses. In contrast, the horse inoculated with RBCs from the acquisition horse tested nPCR positive for *T. haneyi* 15 days after inoculation. It showed parasites in blood smear and developed canonical clinical signs of acute infection.

**Conclusion:**

The findings show that *H. longicornis* ticks cannot transstadially transmit *T. haneyi* to horses.

## 1 Introduction

*Theileria haneyi*, a recently discovered apicomplexan tick-borne hemoparasite, infects horses globally (1–4). Along with *Theileria equi* and *Babesia caballi*, *T. haneyi* is one of the causative agents of equine piroplasmosis, a disease with significant economic implications that affects equids, including horses, donkeys, and zebras. According to the current regulations of the World Organization for Animal Health (formerly Office International des Epizooties—OIE), equine piroplasmosis is a reportable disease that poses severe barriers to animal movement globally (5–7). While countries free from this disease, such as the US, Japan, and New Zealand, enforce strict regulations to prevent the importation of infected horses and to maintain their disease-free status, countries already affected by the disease also limit the import of infected animals to avoid the introduction of potentially diverse and virulent strains.

The severity of *T. haneyi* acute infection is lower than that of *T. equi* and *B. caballi* infections, and clinical signs include hemolytic anemia, fever, lethargy, petechiation, parasitemia, which is indicated by the presence of infected red blood cells (RBCs) in a blood smear, and in some cases, sudden death (3–5). Animals that survive the acute phase of the disease may later develop chronic asymptomatic infection and serve as reservoirs for tick and iatrogenic transmission of the parasite (3–5). It is important to note that vaccines against equine piroplasmosis have not yet been developed. The strategies to monitor and control the infection and disease include the use of molecular and serological diagnostics, and anti-parasitic prophylactic and therapeutic drugs (8–10).

Experimental studies have demonstrated that *T. haneyi* is less virulent to immunocompetent horses than *T. equi* and *B. caballi*. However, the impact of the parasite on high-performance racing horses and working equids is currently unknown (1, 11). Research has shown that *T. haneyi* is resistant to the most commonly used anti-parasitic therapeutic drugs, including imidocarb dipropionate, buparvaquone, tulathromycin, and diclazuril (12, 13). In addition, a previous study has shown that the co-infection of *T. haneyi* and *T. equi* decreases the efficacy of imidocarb dipropionate (14). This decrease in the efficacy of imidocarb dipropionate is highly concerning, given that it is the drug of choice for the treatment of equine piroplasmosis and is widely used for controlling the clinical signs of the disease and curing *T. equi*-infected equids worldwide (8, 9). As *T. haneyi* is considered a novel tick-borne hemoparasite of equids, little is currently known about its lifecycle (1). There are gaps in the literature regarding the knowledge of tick species that are competent in transmitting the parasite to horses.

*Haemaphysalis longicornis*, also known as the Asian longhorned tick, is endemic to East Asia, including countries such as China, Korea, and Japan (15–17). From the Asia-Pacific region, *H. longicornis* has spread to New Zealand and Australia, where it has become a major tick vector for the transmission of the *Theileria orientalis* Ikeda genotype, a hemoparasite with significant economic implications that infects cattle (18–23). *H. longicornis* is a highly aggressive, multihost tick species that can feed on a variety of mammalian hosts (24, 25). As reported in previous studies, it is a competent biological vector for several pathogens that can infect humans and animals, including hemoparasites, bacteria, and viruses (26–32). The invasion of this tick species in the US was confirmed in 2017, and it has been linked to the emerging outbreaks of *T. orientalis* Ikeda infections in cattle across the country (25, 33). Since then, *H. longicornis* has spread to several states in the eastern US, posing a major threat to the country’s public and animal health (34–38).

Considering the ability of *H. longicornis* to transmit the *T. haneyi*-related parasite *T. orientalis* to cattle (27) and given that this tick species feeds efficiently on horses (39, 40), we conducted a controlled experiment to investigate the ability of *H. longicornis* to transstadially transmit *T. haneyi* to horses.

## 2 Materials and methods

### 2.1 Statement of animal care and use approval

All procedures in this study were performed in accordance with the protocols approved by the Institutional Animal Care and Use Committees of the University of Idaho (protocol no. 2041–26) and Washington State University (protocol no. 6982). Furthermore, all procedures adhered to the US National Institutes of Health (NIH) Guide for the Care and Use of Laboratory Animals.

### 2.2 Parasite

In this study, the *T. haneyi* Eagle Pass isolate was used (1). The *T. haneyi* blood stabilate was prepared and horses were infected based on protocols mentioned in previous studies (11–13).

### 2.3 *Haemaphysalis longicornis* ticks

The *H. longicornis* tick colony maintained at the USDA-ARS Animal Disease Research Unit, Pullman, WA, United States, was used in this study. This colony comprised ticks that were collected in the field by Dr. Dana Price (Rutgers University, New Jersey) in New Jersey and kindly provided to the USDA-ARS Animal Disease Research Unit in 2019 for colony rearing. In addition, ticks from this colony had also been used for competence studies in *Theileria* and *Babesia* parasites (27, 41).

### 2.4 *Theileria haneyi* acquisition

**Figure 1:**
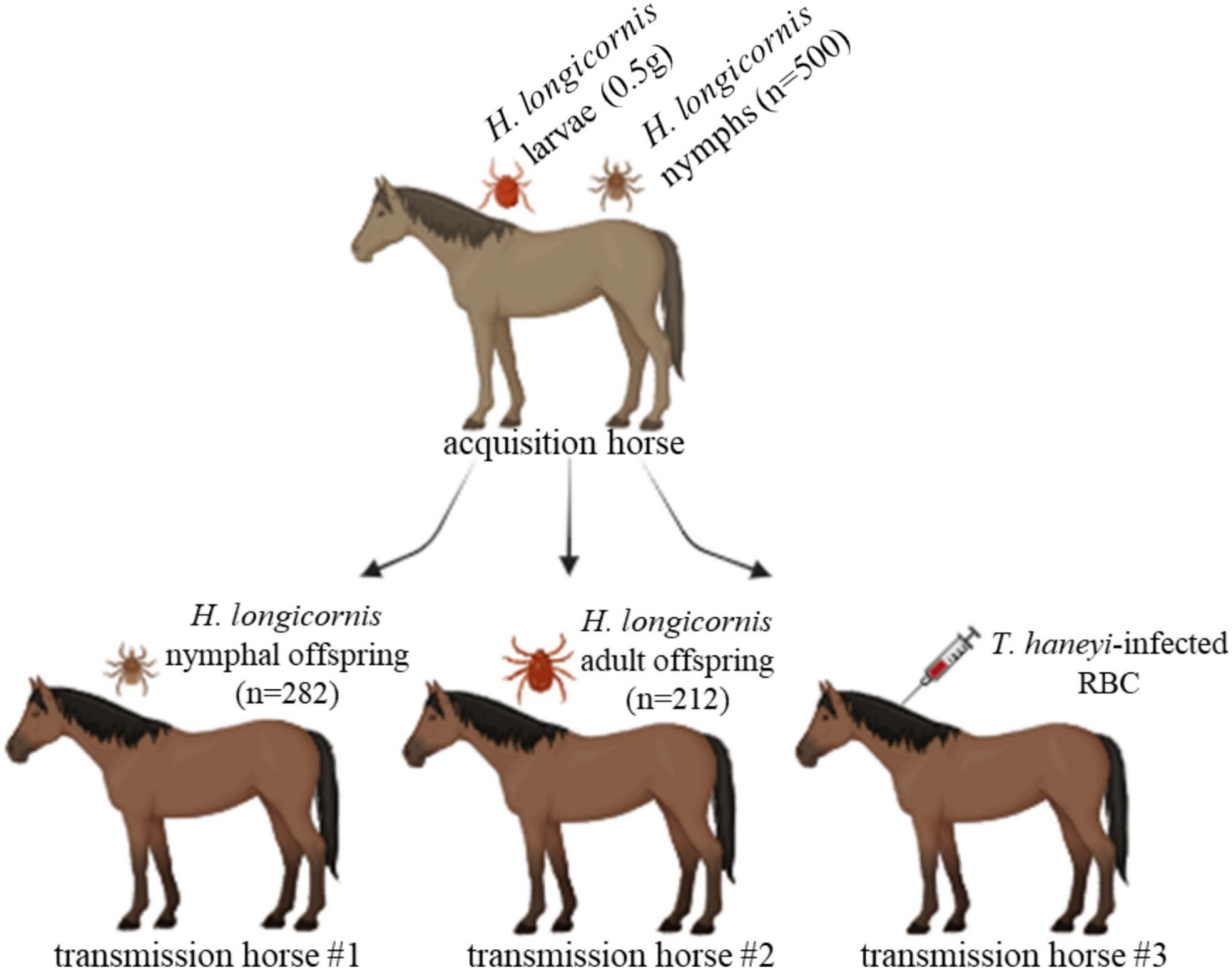
Schematic of the experimental design to investigate the competence of *Haemaphysalis longicornis* to transstadially transmit *Theileria haneyi* to horses. *H. longicornis* larvae (0.5 g) and nymphs (*n* = 500) were fed on a splenectomized *T. haneyi*-infected horse (acquisition horse). Nymphal (*n* = 282) and adult (212) offspring were subsequently fed on naïve horses for transmission (transmission horses 1 and 2, respectively). In addition, red blood cells (RBCs) from the acquisition horse were intravenously inoculated into transmission horse 3. Figure created using BioRender.com.

A Welsh horse chronically infected with the *T. haneyi* Eagle Pass isolate was used for tick parasite acquisition. The horse was infected with *T. haneyi* blood stabilate and monitored throughout the development of chronic infection, as previously described (14). The horse was used as an untreated control in a previous investigation (13). Prior to tick parasite acquisition, the horse was splenectomized to increase parasitemia, which was performed at the Washington State University Veterinary Teaching Hospital (WSU VTH), following standard procedures. Briefly, before the surgery, the horse was kept off feed for 24 h and water for 6–8 h and anesthesia was administered by a veterinary anesthesiologist. A skin incision of approximately 30 cm was made over the sixteenth rib beginning at the paralumbar muscles and extending distally to 5 cm ventral to the costochondral junction. The incision was extended through the subcutaneous tissues and muscles. Then, a careful stab incision was made through the periosteum and peritoneum. The apex of the spleen was exteriorized, exposing the gastrosplenic ligament and associated vessels, which were ligated and transected. The spleen was partially elevated and shifted ventrally to access the nephrosplenic ligament. The ligament was bluntly and sharply dissected off the axial splenic surface. Then, phenylephrine was injected directly into the spleen to cause splenic contraction. The hilus was double-ligated and cauterized, and the spleen was removed. Post-surgery recovery followed standard procedures at WSU VTH. Post-surgery pain was managed by the administration of non-steroidal anti-inflammatory drugs at recommended dosages. The horse was discharged from WSU VTH 1 week after the surgery. It was monitored for parasitemia by Giemsa staining of blood smear followed by microscopy analysis, *T. haneyi* nPCR, and evaluation of clinical signs of equine piroplasmosis. Fourteen days after splenectomy, when evident signs of parasite replication were observed in peripheral blood, the acquisition horse was infested with 0.5 g of unfed *H. longicornis* larvae and 500 unfed, freshly molted *H. longicornis* nymphs. The ticks were placed under separate cloth patches on the acquisition horse (Figure 1). Once the larvae and nymphs fed to repletion, they were collected, placed in the incubator for molting, and used for transmission steps. Furthermore, blood from the acquisition horse was collected on day 3 post-tick application. The blood was washed to remove leukocytes, and RBCs were suspended in one volume of Puck’s Saline G (Fisher Scientific) with 20% polyvinylpyrrolidone (PVP-40) (Sigma-Aldrich) and frozen in liquid nitrogen until use.

### 2.5 *Theileria haneyi* transmission

Freshly molted, unfed nymphs and adults, which were the offspring of the acquisition larvae and nymphs, respectively, were placed on two naïve, spleen-intact horses for transmission (Figure 1). A total of 282 nymphs and 212 adults were placed under a cloth patch on transmission horses 1 and 2, respectively (Figure 1). After 3 days of feeding, 10 partially fed nymphs and 10 partially fed adults were collected for PCR analysis. DNA extracted from whole individual partially fed nymphs and salivary glands (SGs) dissected from individual adults was used for this analysis. The remaining ticks fed to repletion. After tick feeding, both horses were monitored for 38 days for the presence of *T. haneyi* DNA in peripheral blood and signs of acute infection. In addition, 1 mL of RBCs from the acquisition horse was intravenously inoculated into another horse (transmission horse 3) (Figure 1). After RBC inoculation, transmission horse 3 was monitored for 30 days to detect *T. haneyi* DNA in peripheral blood and signs of acute infection.

### 2.6 Blood cell count

The blood cell count of the acquisition and transmission horses was evaluated using the ProCyte One™ Hematology Analyzer (IDEXX Laboratories, Inc., Westbrook, ME, United States) in accordance with the manufacturer’s protocol. Briefly, peripheral blood was collected into Vacutainer^®^ tubes containing EDTA (BD Company, Franklin Lakes, NJ, United States) at several time points post-splenectomy, post-tick infestation, and post-RBC inoculation. The collected whole-blood samples were mixed for approximately 5 min, and the number of total leukocytes, lymphocytes, monocytes, neutrophils, and RBCs was measured. White blood cell (WBC) populations were counted as 1,000 cells/mL of blood, and RBC populations were counted as 1,000,000 cells/mL of blood.

### 2.7 *Theileria haneyi* nested PCR

The presence of *T. haneyi* DNA in the acquisition and transmission horses and in the acquisition and transmission ticks was monitored using nested PCR (nPCR) targeting a hypothetical gene (GenBank accession number: MT896770.1), syntenic to the *T. equi ema-1* gene, as described in previous studies (1, 12, 13). Briefly, DNA extraction was performed on whole-blood and tick samples using the Qiagen DNeasy^®^ Blood & Tissue Kit (Qiagen, Germantown, MD, United States) in accordance with the manufacturer’s instructions. As positive and negative controls for nPCR, DNA obtained from a horse that had consistently tested positive for *T. haneyi* and sterile 1X phosphate-buffered saline (PBS) (Thermo Fisher Scientific, Waltham, MA. United States) were used, respectively, as described in previous studies (1, 2, 12, 13, 42).

### 2.8 *Haemaphysalis longicornis* GAPDH PCR

The presence of amplifiable DNA in the tick samples, including larvae, nymphs, and adult SG, was evaluated using PCR by employing specific primers for the *H. longicornis* GAPDH gene (GenBank accession number: MW148932.1). Briefly, DNA from individual larvae, nymphs, and SG was extracted using the Qiagen DNeasy^®^ Blood & Tissue Kit in accordance with the manufacturer’s instructions. Of each sample, 2 ul was used for PCR with 1 μM of each of the following primers: 5-tgggcgcggagtacgtggt-3 and 5-cgtgcacggtggacatgagg-3. To achieve an amplicon size of 270 bp, DreamTaq green PCR Master Mix (Thermo Fisher Scientific) was used with an annealing temperature of 55°C. The amplicon was analyzed using 1.5% agarose gel, and ChemiDoc™ Touch Imaging System (Bio-Rad, Hercules, CA, United States) was used for image acquisition and subsequent visualization. In addition, the *H. longicornis* GAPDH amplicon was sequenced for confirmation.

### 2.9 Horse β-actin PCR

Horse blood samples were used for DNA extraction as described earlier. The samples were subjected to PCR using specific primers for equine β-actin (GenBank accession number: XM_023655002.1) to demonstrate the presence of amplifiable DNA. Of each sample, 2 μl was used for PCR with 1 μM of each of the following primers: 5-tggcatccacgaaactacct-3 and 5-tctgctggaaggtggacaat-3. To achieve an amplicon size of 248 bp, DreamTaq green PCR Master Mix (Thermo Fisher Scientific) was used with an annealing temperature of 55°C. The amplicon was analyzed using 1.5% agarose gel, and ChemiDoc™ Touch Imaging System (Bio-Rad) was used for image acquisition and subsequent visualization. In addition, the horse β-actin amplicon was sequenced for confirmation.

## 3 Results

### 3.1 *Theileria haneyi* infection in the splenectomized horse during tick acquisition

**Figure 2:**
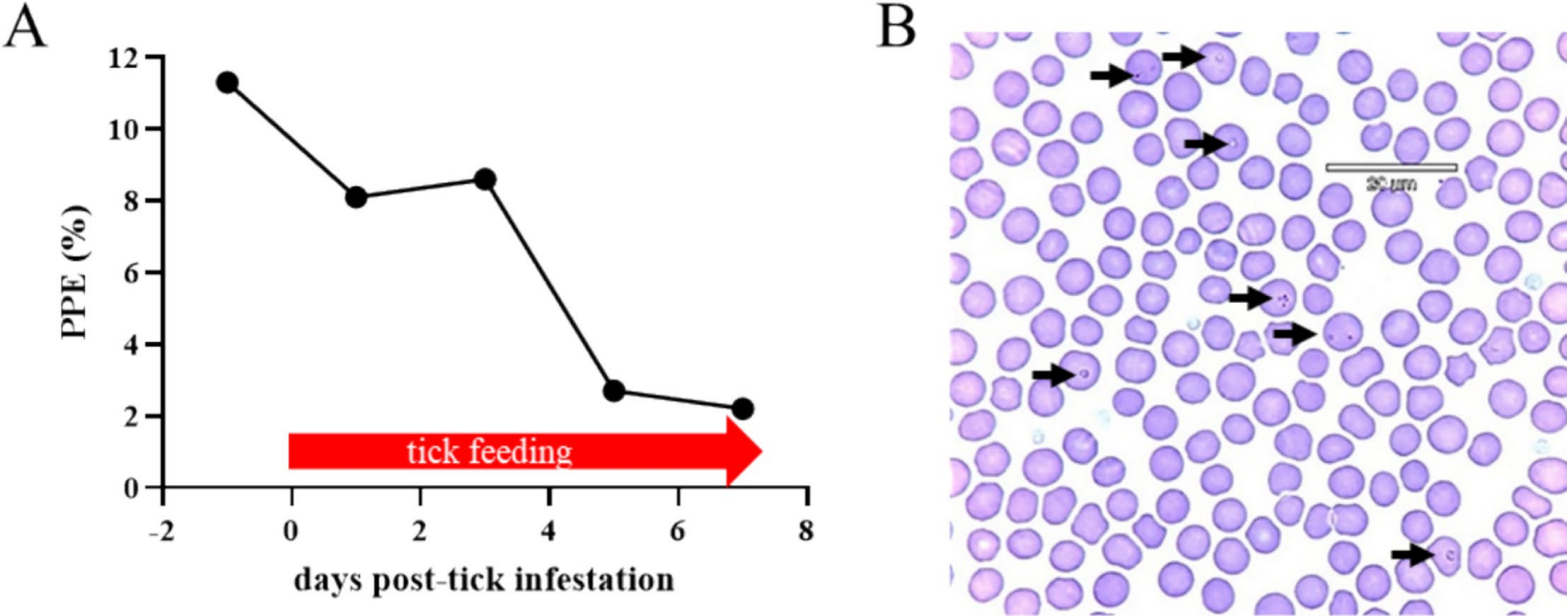
Percentage of parasitized erythrocytes (PPE) during the larval and nymphal *Haemaphysalis longicornis* feeding period on the acquisition horse **(A)**. A representative blood smear Giemsa staining. Arrows indicate *Theileria haneyi*-infected erythrocytes **(B)**.

**Figure 3:**
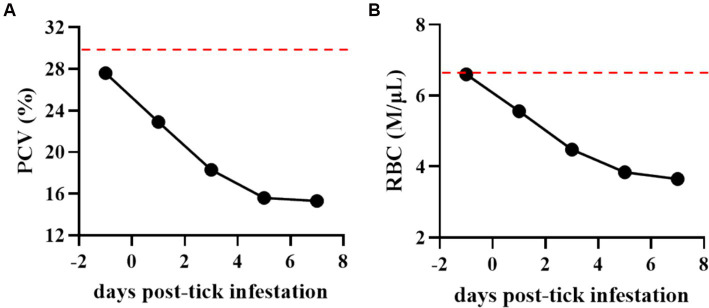
Percentage of packed cell volume (PCV) **(A)** and total number of red blood cells (RBCs) **(B)** in the *Theileria haneyi*-infected acquisition horse during tick infestation. Dashed red lines represent the lower physiological limit for PCV and total RBCs in equines.

**Figure 4:**
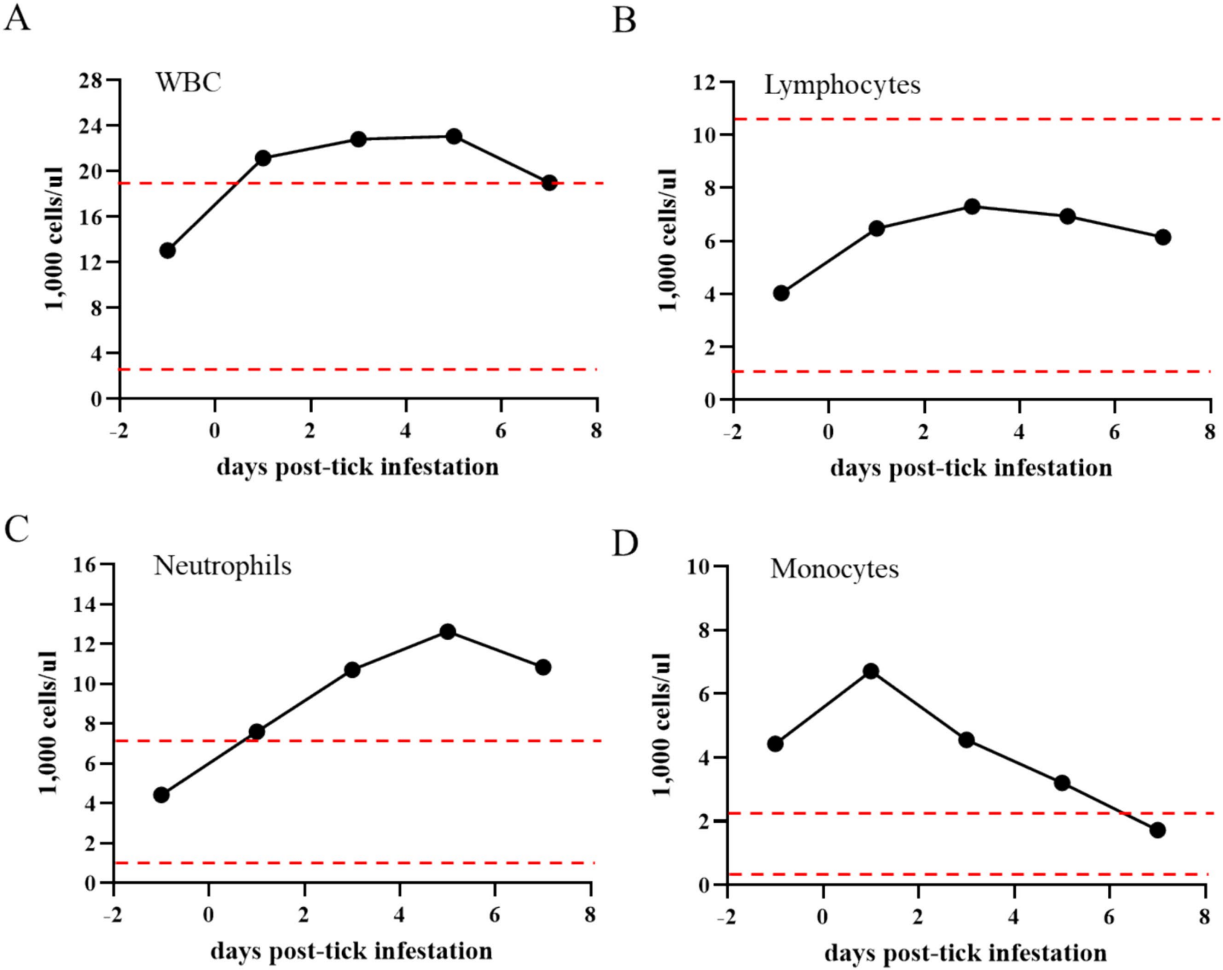
Total number of white blood cells (WBCs) **(A)**, lymphocytes **(B)**, neutrophils **(C)**, and monocytes **(D)** in the *Theileria haneyi*-infected acquisition horse during tick infestation. Dashed red lines represent the upper and lower physiological limits for each cell population in equines.

To investigate the competence of *H. longicornis* in acquiring and transmitting *T. haneyi*, an infected horse (i.e., the acquisition horse) with a high percentage of parasitized erythrocytes (PPE) was selected for tick acquisition. This chronically *T. haneyi*-infected horse was subjected to splenectomy and monitored for PPE and clinical signs of acute infection (Figures 2–4). Fourteen days after splenectomy, ticks were placed on the acquisition horse. During the tick feeding period, variations in PPE were observed, 8.1, 8.6, 2.7, and 2.2% on days 1, 3, 5, and 7 post-infestation, respectively (Figure 2A). The presence of *T. haneyi*-infected erythrocytes was observed in blood smear via Giemsa staining. A representative blood smear staining from the acquisition horse is shown in Figure 2B. In addition, peripheral blood of the acquisition horse was collected on day 3 post-tick infestation and used for transmission.

During the tick feeding period, packed cell volume (PCV), total number of RBCs, and complete blood cell count were examined (Figure 3). PCV decreased from 27.6% on day 0 when ticks were placed on the animal to 15.3% on day 7 when all ticks had dropped. Notably, it was below the physiological values for equines, decreasing by more than 50% during the tick feeding period (Figure 3A). Similarly, the total number of RBCs decreased by nearly 50% (from 6.6 to 3.65 M/μL) during tick feeding (Figure 3B). The changes in the peripheral blood cells of the acquisition horse during the tick feeding period are shown in Figure 4. The total number of WBCs was increased above the physiological level for equines during days 1 to 7 post-tick infestation (Figure 4A). This increase was not due to the change in the number of lymphocytes as it was within the normal levels during the period (Figure 4B). Interestingly, the levels of both neutrophils and monocytes were markedly increased (Figures 4C,D). All these observations on parasitemia and blood cell alterations show that *H. longicornis* larvae and nymphs fed on the acquisition horse during the development of high levels of *T. haneyi* PPE and canonical signs of acute parasite infection.

### 3.2 Acquisition feeding of *Haemaphysalis longicornis* larvae and nymphs on the *Theileria haneyi*-infected, splenectomized horse

**Figure 5:**
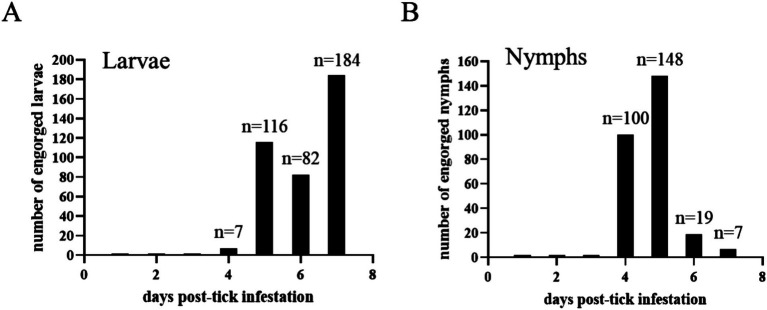
The number of engorged *Haemaphysalis longicornis* larvae **(A)** and nymphs **(B)** collected every day post-infestation on the acquisition horse.

A total of 7, 116, 82, and 70 engorged larvae were collected from the acquisition horse on days 4, 5, 6, and 7 post-infestation, respectively (Figure 5A). Regarding nymphs, a total of 100, 148, 19, and 1 engorged ticks were collected from the acquisition horse on days 4, 5, 6, and 7 post-infestation, respectively (Figure 5B). Engorged larvae and nymphs were kept in an incubator for molting and hardening and used for the transmission phase of the study.

A portion of the engorged larvae and nymphs that fed on the acquisition horse, whose number decreased between days 4 and 6 post-infection, was evaluated using nPCR for the presence of *T. haneyi* DNA (Table 1). The results of the nPCR conducted with DNA extracted from 10 individual engorged larvae showed the presence of *T. haneyi* DNA in all ticks tested. Interestingly, the presence of parasite DNA was detected in only 4 of 10 engorged nymphs measured. In the PCR analysis, the *H. longicornis* GAPDH gene consistently showed the presence of amplifiable tick DNA in all samples analyzed (Table 1). Altogether, these observations show that a representative number of larvae and nymphs fed to repletion on the acquisition horse. Furthermore, nPCR results showed the presence of *T. haneyi* DNA in the engorged larvae and nymphs.

**Table 1:** Results of *Theileria haneyi* nPCR and *Haemaphysalis longicornis* GAPDH PCR in 10 acquisition larvae (L1–10) and 10 acquisition nymphs (N1–10).

	L1	L2	L3	L4	L5	L6	L7	L8	L9	L10
*T. haneyi* nPCR	+	+	+	+	+	+	+	+	+	+
*H. longicornis* GAPDH PCR	+	+	+	+	+	+	+	+	+	+

	N1	N2	N3	N4	N5	N6	N7	N8	N9	N10
*T. haneyi* nPCR	−	−	−	−	+	+	−	+	+	−
*H. longicornis* GAPDH PCR	+	+	+	+	+	+	+	+	+	+

(−) negative PCR result; (+) positive PCR result.

### 3.3 Transmission feeding of *Haemaphysalis longicornis* nymphs and adults on naïve horses

Freshly molted *H. longicornis* nymphs (*n* = 282) and adults (212), offspring of *T. haneyi* acquisition larvae and nymphs, respectively, were placed on transmission horses 1 and 2, respectively, to investigate the transmission of the tick parasite (Figure 1). To examine the presence of *T. haneyi* DNA, 10 partially fed nymphs and 10 partially fed adults were collected for nPCR on day 3 of feeding. The results of nPCR using individual nymphs and individual SG dissected from adult ticks showed no evidence of the presence of *T. haneyi* DNA in the transmission ticks (Table 2). The presence of amplifiable DNA in the tick samples was confirmed by the PCR of the *H. longicornis* GAPDH gene (Table 2). A total of 79 replete nymphs were recovered from transmission horse 1, over 8 days of feeding, while 65 replete adults were obtained from transmission horse 2, over 10 days of feeding.

**Table 2:** Results of *Theileria haneyi* nPCR and *Haemaphysalis longicornis* GAPDH PCR in 10 transmission nymphs (N1–10) and 10 individual salivary glands (SG1–10) dissected from adult transmission ticks.

	N1	N2	N3	N4	N5	N6	N7	N8	N9	N10
*T. haneyi* nPCR	−	−	−	−	−	−	−	−	−	−
*H. longicornis* GAPDH PCR	+	+	+	+	+	+	+	+	+	+

	SG1	SG2	SG3	SG4	SG5	SG6	SG7	SG8	SG9	SG10
*T. haneyi* nPCR	−	−	−	−	−	−	−	−	−	−
*H. longicornis* GAPDH PCR	+	+	+	+	+	+	+	+	+	+

(−) negative PCR result; (+) positive PCR result.

Transmission horses 1 and 2 were monitored for 38 days after tick infestation for the presence of *T. haneyi* DNA in peripheral blood, changes in blood cell count, and signs of acute infection. *T. haneyi* nPCR results showed no evidence of the parasite DNA in the peripheral blood of the transmission horses (Supplementary Tables S1, S2). Both horses’ PCV values remained within the normal range, except when the PCV of transmission horse 1 fluctuated from 28 to 29% between days 10 and 22 post-infestation and when the PCV of transmission horse 2 decreased to 22.9% on day 20 post-infestation. After these fluctuations, PCV values returned to normal levels (Supplementary Tables S1, S2). No significant increase in body temperature was observed in the transmission horses during the post-infestation monitoring period. In addition, no significant changes in the absolute number of total WBCs, lymphocytes, neutrophils, and monocytes were observed during the monitoring period (Supplementary Tables S1, S2). Together, these results demonstrate no evidence of *T. haneyi* transstadial transmission by *H. longicornis* nymphs and adults.

### 3.4 Blood transmission of *Theileria haneyi* to a naïve horse

To investigate the presence of viable, infectious *T. haneyi* parasites in the blood of the acquisition horse, its RBCs were inoculated into a naïve horse (transmission horse 3) (Figure 1). After inoculation, transmission horse 3 was monitored for 30 days for signs of acute infection (Figure 1). PCR results showed the presence of *T. haneyi* DNA in its peripheral blood, starting from day 15 post-inoculation (Table 3). Subsequently, on day 21 post-inoculation, parasites were visualized using microscopy of blood smear. The presence of amplifiable DNA extracted from peripheral blood samples of transmission horse 3 was confirmed using endpoint PCR of the horse β-actin gene (Table 3).

**Table 3:** Results of *Theileria haneyi* nPCR, blood smear, and horse β-actin PCR in transmission horse 3 infected with blood from the transmission animal.

DPI																
	1	3	5	7	9	11	13	15	17	19	21	23	25	27	29	30
*T. haneyi* nPCR	−	−	−	−	−	−	−	+	+	+	+	+	+	+	+	+
Parasite in blood smear	−	−	−	−	−	−	−	−	−	−	+	+	+	+	+	+
Horse β-actin PCR	+	+	+	+	+	+	+	+	+	+	+	+	+	+	+	+

DPI, days post-infection; (−) negative PCR result; (+) positive PCR result.

**Figure 6:**
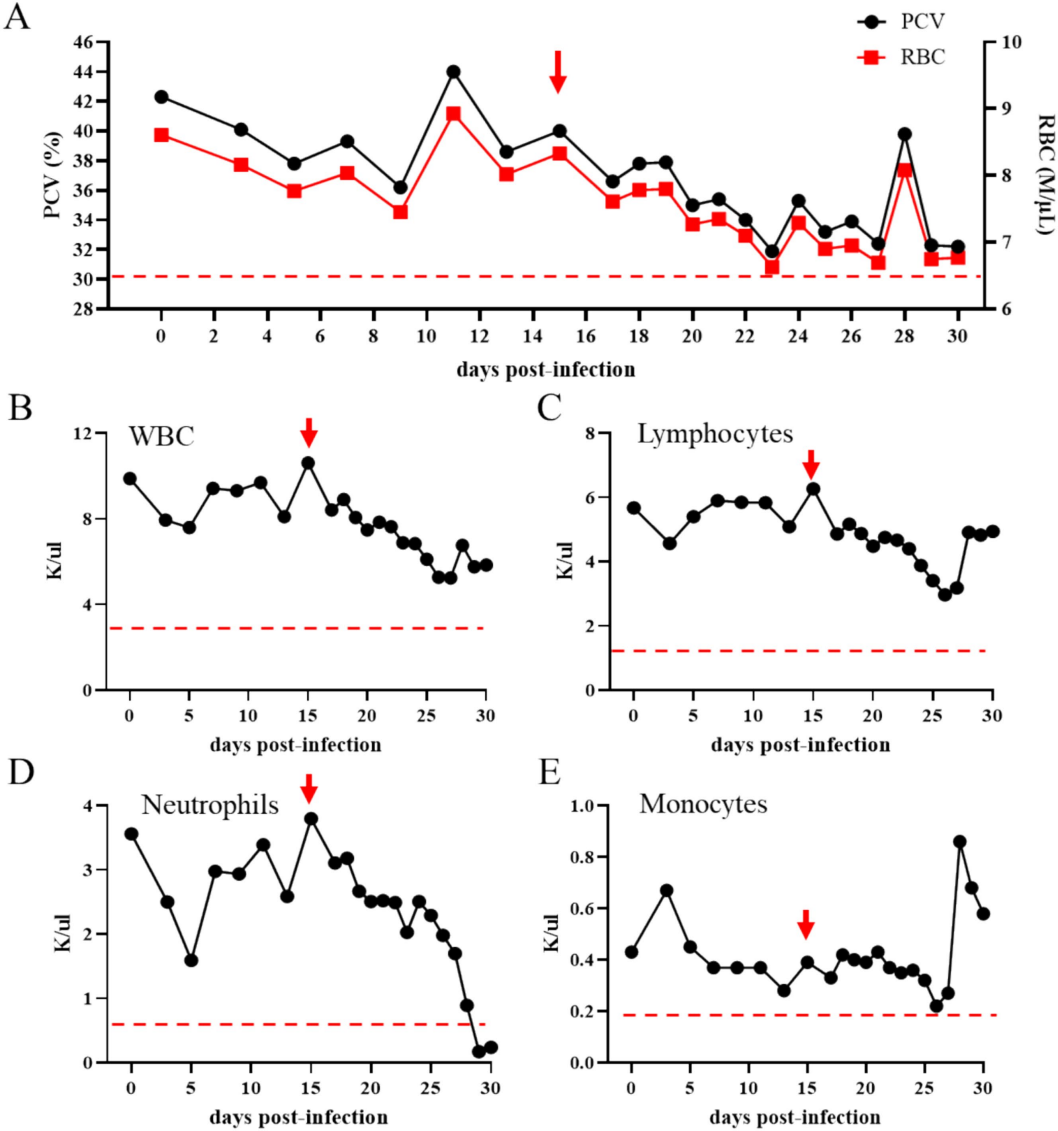
Percentage of packed cell volume (PCV) (**A**; the left *y*-axis) and total number of red blood cells (RBCs) (**A**; the right *y*-axis) in transmission horse 3 following inoculation of RBCs from the *Theileria haneyi* acquisition horse. Panels **(B–E)** present the absolute numbers of white blood cells (WBCs), lymphocytes, neutrophils, and monocytes in peripheral blood, respectively, of transmission horse 3 following inoculation of RBCs from the acquisition horse. Red arrows indicate the time point that the animal became nPCR positive for *T. haneyi*. Dashed red lines indicate the lowest physiological levels for PCV and RBCs **(A)**, WBCs **(B)**, lymphocytes **(C)**, neutrophils **(D)**, and monocytes **(E)**.

*Theileria haneyi* infection in transmission horse 3 was also confirmed by a marked decrease in PCV and total RBCs in peripheral blood, starting from day 15 post-inoculation (Figure 5A). The total number of WBCs and leukocytes showed a tendency to decrease after inoculation; however, the levels remained within the normal range for equines (Figures 6B,C). In addition, the total number of neutrophils in peripheral blood decreased below the physiological levels on days 29 and 30 post-inoculation (Figure 6D). Two marginal peaks in the number of monocytes were observed on day 3 and day 28 post-inoculation, but these levels were within the physiological range. Taken together, the results of PCR, blood smear analysis, and changes in blood cell counts demonstrated that transmission horse 3 was infected with *T. haneyi* following inoculation with RBCs from the acquisition horse (Figure 6).

## 4 Discussion

Studies investigating the competence of tick species in transmitting both endemic and exotic tick-borne pathogens are crucial for developing and implementing effective control strategies to protect human, livestock, and wildlife health. Such studies are especially important in light of the adaptation of arthropods to diverse environments and global interconnectivity, which significantly affect tick populations and their habitats. The recent prevalence of *H. longicornis* in the eastern USA represents a serious threat to public and animal health (25, 34, 35, 38, 43). *H. longicornis* is a major vector for several pathogens, including *Anaplasma*, *Babesia*, *Borrelia*, *Ehrlichia*, *Rickettsia*, *Theileria*, and numerous viruses, which affect several mammalian hosts (18, 44–46). This pathogen-transmitting nature of *H. longicornis* is attributable to important aspects of its lifecycle, such as its multihost characteristic and remarkable adaptability to environmental conditions (47, 48). It has been recently demonstrated that *H. longicornis* is a competent vector for transmitting the US isolate of *T. orientalis* genotype Ikeda to cattle, posing a serious risk to the country’s bovine industry (27, 33, 49).

This study examined *T. haneyi*, a recently discovered tick-borne hemoparasite of equids, and investigated the ability of *H. longicornis* to transstadially transmit this parasite to horses. *H. longicornis* larvae and nymphs were used for parasite acquisition on a splenectomized infected horse, and their respective offspring (nymphs and adults) were evaluated for parasite transmission to naïve hosts. The ticks fed efficiently to repletion on the acquisition horse, which showed high PPE during the feeding period. Engorged larvae and nymphs molted successfully into nymphs and adults, respectively, which were then used for parasite transmission. *H. longicornis* nymphs and adults were placed on naïve horses (transmission horses) to investigate *T. haneyi* transmission. Partially fed transmission nymphs and adults tested negative for nPCR for the presence of *T. haneyi* DNA, and the transmission horses remained healthy and tested nPCR negative for *T. haneyi* during the observation period. The transmission horses were monitored for 38 days post-tick infestation, which is more than twice the average prepatent time for the *T. haneyi*-related parasite *T. equi* to show signs of infection in horses following tick infestation (3–5, 50). The results of this controlled experiment showed no evidence of transstadial transmission of *T. haneyi* to horses via *H. longicornis*. In contrast, a naïve horse inoculated with RBCs from the acquisition horse developed canonical signs of acute *T. haneyi* infection, including anemia, the presence of parasites in blood smear, and nPCR-positive results for parasite DNA.

The observations on the acquisition horse reinforce that the spleen is a major immunological site for controlling parasite replication during the chronic phase of infection with apicomplexan hemoparasites (14, 51, 52). In the present study, the removal of the spleen in the chronically infected acquisition horse aggravated the infection, which was characterized by increased parasitemia in peripheral blood, a decrease in the total number of RBCs, and a decrease in PCV, consistent with previous studies (1, 14, 53). The spleen-intact transmission horse 3 that received RBCs from the acquisition horse showed the presence of parasite DNA, starting from day 15 post-inoculation, and developed mild but canonical signs of acute infection, which is also in line with previous studies (1, 11).

Tick vector competence is the ability of a specific tick species to acquire, maintain, and transmit a pathogen (54, 55). It is a complex process involving multiple factors related to ticks, pathogens, and susceptible hosts. First, ticks should efficiently feed on an infected host to acquire the pathogen. Second, they should maintain the pathogen through molting and transmit it by injecting their saliva during feeding on a naïve host. This process becomes even more complex with apicomplexan hemoparasites, such as *Theileria* spp. and *Babesia* spp., which undergo sexual reproduction during their stages in competent tick vectors (5, 56, 57). Moreover, *H. longicornis* ticks efficiently feed on a wide range of mammalian hosts, including humans and domestic and wildlife animals (23, 48). Our results reinforce this observation by showing that *H. longicornis* larvae, nymphs, and adults feed efficiently on horses, regardless of the equine status (either infected or naïve). Irrespective of the efficient feeding of all *H. longicornis* stages on horses, our results indicate that this tick species is not competent in transstadially transmitting *T. haneyi* to horses. Our findings showed the presence of *T. haneyi* DNA in all 10 tested acquisition larvae, whereas only 4 of the 10 analyzed acquisition nymphs tested positive for the parasite DNA. This finding is interesting considering that both larvae and nymphs fed simultaneously on a horse with *T. haneyi* parasitemia ranged from 8% down to 2% during the tick feeding period. It was beyond the scope of this study to investigate the tick and/or parasite factors associated with the ability of *H. longicornis* to transmit some *Theileria* species, such as *T. orientalis*, but not others, such as *T. haneyi*, which deserves further studies.

*Theileria haneyi* is genetically closely related to *T. equi*, with both hemoparasites infecting equids and causing similar clinical signs during acute infection, including hemolytic anemia, fever, anorexia, and prostration (1, 5, 11, 14). However, there are marked differences between these two parasites. Whereas experimental studies have shown that *T. haneyi* is only mildly virulent to immunocompetent horses, experimental and field data show that *T. equi* causes severe anemia and fever and can be lethal in some cases (1, 5, 11, 14). In addition, *T. haneyi* is resistant to imidocarb dipropionate, the drug of choice for treating equine piroplasmosis, whereas *T. equi* is susceptible to this drug, with only sporadic cases of resistance being reported (4, 8, 9, 53). Experimental studies have demonstrated that *Rhipicephalus microplus* ticks are competent vectors for transmitting *T. equi* (58, 59). However, given that *R. microplus* are monoxenic ticks and that horses are not natural hosts to this species, the epidemiological implications of this finding remain to be determined. Currently, no information is available on whether *R. microplus* is competent in transmitting *T. haneyi*. Therefore, considering these biological differences between *T. haneyi* and *T. equi*, which specific characteristics would allow or prevent these parasites from being transmitted by a particular tick species remains unknown. Thus, although in the present study, we showed that *H. longicornis* is not a competent vector for the transstadial transmission of *T. haneyi*, whether this tick species is a competent vector for *T. equi* remains to be determined. Furthermore, the present findings do not rule out the possibility that as *H. longicornis* becomes more widespread and adapted to new environments, such as the eastern, northern, and southern USA, it may acquire the capacity to transmit *T. haneyi* to equids, as well as new pathogens to humans, livestock, and wildlife.

Considering the recent invasion of *H. longicornis* into the eastern USA and the wide host range of this tick species, how this invasion will affect the resident tick populations and the dynamics of tick-borne diseases in humans, livestock, and wildlife remains to be determined. Without doubt, this is a multifaceted scenario that could affect already endemic diseases, such as Lyme disease (60), and facilitate the emergence of previously exotic pathogens, such as the recent outbreaks of *T. orientalis* Ikeda in the USA (27, 33, 34, 38). In the present study, we used 0.5 g of larvae and 500 nymphs for acquisition and 282 and 212 nymphal and adult offspring, respectively, for transmission. Our findings on tick infection status and animal health showed no evidence for the transstadial transmission of *T. haneyi* by *H. longicornis*. Although this raises several questions about the biological factors that prevent *H. longicornis* from being a competent vector for *T. haneyi*, these findings can be reassuring for stakeholders in the equine industry, considering that equids may not be at risk of infection with this *Theileria* species due to *H. longicornis* infestation. However, this conclusion is based on a controlled experiment, and constant interactions between ticks, pathogens, and mammalian hosts, including equids, may change this scenario. Therefore, maintaining robust livestock and wildlife animal health surveillance programs is essential to control tick-borne pathogens, particularly *T. haneyi*, *T. equi*, and *B. caballi*, the causative agents of equine piroplasmosis.

## 5 Conclusion

In conclusion, the findings of the present study demonstrated that *H. longicornis* cannot transstadially transmit the *T. haneyi* Eagle Pass isolate to horses. Although the acquisition horse developed high *T. haneyi* parasitemia in peripheral blood during tick acquisition and parasite DNA was detected in larval and nymphal acquisition ticks, no evidence was found for the competence of *H. longicornis* to transmit *T. haneyi* to horses.

## Data Availability

The datasets presented in this study can be found in online repositories. The names of the repository/repositories and accession number(s) can be found in the article/Supplementary material.

## Supplementary material

SUPPLEMENTARY TABLE S1 Transmission horse 1.

SUPPLEMENTARY TABLE S2 Transmission horse 2.
